# Perspectives of European Patient Advocacy Groups on Volunteer Registries and Vaccine Trials: VACCELERATE Survey Study

**DOI:** 10.2196/47241

**Published:** 2024-04-04

**Authors:** Sophia Themistocleous, Christos D Argyropoulos, Paris Vogazianos, George Shiamakkides, Evgenia Noula, Andria Nearchou, Andreas Yiallouris, Charalampos Filippou, Fiona A Stewart, Markela Koniordou, Ioannis Kopsidas, Helena H Askling, Sirkka Vene, Amandine Gagneux-Brunon, Jana Baranda Prellezo, Elena Álvarez-Barco, Jon Salmanton-García, Janina Leckler, Alan J Macken, Ruth Joanna Davis, Anna Maria Azzini, Charis Armeftis, Margot Hellemans, Romina Di Marzo, Catarina Luis, Ole F Olesen, Olena Valdenmaiier, Stine Finne Jakobsen, Pontus Nauclér, Odile Launay, Patrick Mallon, Jordi Ochando, Pierre van Damme, Evelina Tacconelli, Theoklis Zaoutis, Oliver A Cornely, Zoi Dorothea Pana

**Affiliations:** 1 School of Medicine European University Cyprus Nicosia Cyprus; 2 Department of Behavioural and Social Sciences School of Humanities, Social and Education Sciences European University Cyprus Nicosia Cyprus; 3 Faculty of Medicine and University Hospital Cologne Institute of Translational Research, Cologne Excellence Cluster on Cellular Stress Responses in Aging-Associated Diseases (CECAD) University of Cologne Cologne Germany; 4 Faculty of Medicine University Hospital Cologne, Department I of Internal Medicine, Center for Integrated Oncology Aachen Bonn Cologne Duesseldorf (CIO ABCD) and Excellence Center for Medical Mycology (ECMM) University of Cologne Cologne Germany; 5 Collaborative Center for Clinical Epidemiology and Outcomes Research (CLEO) Athens Greece; 6 Department of Infectious Diseases Karolinska University Hospital Stockholm Sweden; 7 Division of Infectious Diseases Department of Medicine, Solna Karolinska Institutet Stockholm Sweden; 8 Centre d’investigation clinique-INSERM 1408 University Hospital of Saint-Etienne Saint-Etienne France; 9 Groupe Immunité Muqueuse et Agents Pathogènes (GIMAP) EA3064 – Medical School of Saint-Etienne University of Lyon Saint-Etienne France; 10 Microbiology Section, Department of Pharmaceutical Sciences and of Health Faculty of Pharmacy Universidad San Pablo-Centro de Estudios Universitarios (CEU) Madrid Spain; 11 Centre for Experimental Pathogen Host Research University College Dublin School of Medicine National University of Ireland Dublin Ireland; 12 Department of Diagnostic and Public Health Infectious Diseases University of Verona Verona Italy; 13 Vaccine & Infectious Disease Institute - VAXINFECTIO, Centre of Evaluation of Vaccination, Faculty of Medicine and Health Science Universiteit Antwerpen Antwerp Belgium; 14 European Vaccine Initiative (EVI) Heidelberg Germany; 15 Centre of Excellence for Health, Immunity and Infections (CHIP) Rigshospitalet University of Copenhagen Copenhagen Denmark; 16 Inserm CIC 1417, I-REIVAC University Hospital of Cochin-Broca-Hôtel-Dieu University of Paris-Descartes Paris France; 17 Faculty of Medicine and University Hospital Cologne Clinical Trials Centre Cologne (ZKS Köln) University of Cologne Cologne Germany; 18 German Centre for Infection Research (DZIF) Partner Site Bonn-Cologne Cologne Germany; 19 Faculty of Medicine, and University Hospital Cologne Center for Molecular Medicine Cologne (CMMC) University of Cologne Cologne Germany

**Keywords:** patient advocacy groups, clinical trials, volunteer registry, vaccines, public health, healthcare, COVID-19, vaccine trial, VACCELERATE, health promotion, health advocate, clinical trial

## Abstract

**Background:**

The VACCELERATE Pan-European Scientific network aims to strengthen the foundation of vaccine trial research across Europe by following the principles of equity, inclusion, and diversity. The VACCELERATE Volunteer Registry network provides access to vaccine trial sites across the European region and supports a sustainable volunteer platform for identifying potential participants for forthcoming vaccine clinical research.

**Objective:**

The aim of this study was to approach members of patient advocacy groups (PAGs) across Europe to assess their willingness to register for the VACCELERATE Volunteer Registry and their perspectives related to participating in vaccine trials.

**Methods:**

In an effort to understand how to increase recruitment for the VACCELERATE Volunteer Registry, a standardized survey was developed in English and translated into 8 different languages (Dutch, English, French, German, Greek, Italian, Spanish, and Swedish) by the respective National Coordinator team. The online, anonymous survey was circulated, from March 2022 to May 2022, to PAGs across 10 European countries (Belgium, Cyprus, Denmark, France, Germany, Greece, Ireland, Italy, Spain, and Sweden) to share with their members. The questionnaire constituted of multiple choice and open-ended questions evaluating information regarding participants’ perceptions on participating in vaccine trials and their willingness to become involved in the VACCELERATE Volunteer Registry.

**Results:**

In total, 520 responses were collected and analyzed. The PAG members reported that the principal criteria influencing their decision to participate in clinical trials overall are (1) the risks involved, (2) the benefits that will be gained from their potential participation, and (3) the quality and quantity of information provided regarding the trial. The survey revealed that, out of the 520 respondents, 133 individuals across all age groups were “positive” toward registering in the VACCELERATE Volunteer Registry, with an additional 47 individuals reporting being “very positive.” Respondents from Northern European countries were 1.725 (95% CI 1.206-2.468) times more likely to be willing to participate in the VACCELERATE Volunteer Registry than respondents from Southern European countries.

**Conclusions:**

Factors discouraging participants from joining vaccine trial registries or clinical trials primarily include concerns of the safety of novel vaccines and a lack of trust in those involved in vaccine development. These outcomes aid in identifying issues and setbacks in present registries, providing the VACCELERATE network with feedback on how to potentially increase participation and enrollment in trials across Europe. Development of European health communication strategies among diverse public communities, especially via PAGs, is the key for increasing patients’ willingness to participate in clinical studies.

## Introduction

Within the VACCELERATE consortium, the VACCELERATE Volunteer Registry was assembled on the principles of equity, inclusion, and diversity in vaccine clinical research across Europe [1-3]. Patient advocacy groups (PAGs) personify all 3 principles, with the aim of increasing community participation in research [4-6]. PAG members can monitor the latest news in medical conduct, have access to hard-to-reach medical procedures and treatments, and communicate with health care professionals [5]. Patients with concomitant chronic conditions are largely excluded from three-quarters of research studies listed in ClinicalTrials.gov [7]. Hence, the mission of PAGs is to assist with attaining diversity and equal representation of populations in trials, especially with patients with comorbidities who are more prone to be severely affected by infections [8], as well as overcome other barriers, such as language and limited health education. For example, the European Patients Forum operates to make patient organization voices heard and contends with policy-formulating processes affecting patients across Europe [9]. Alternatively, the European Patients’ Academy on Therapeutic Innovation acts to engage patients through their Patient Expert Training Programme that operates to educate and train in the context of medicine development and research [9]. These initiatives raise patients’ awareness and health literacy and may alleviate concerns regarding clinical trial research.

Another factor is trust in the health care system and its professionals. Health care providers expressing a level of optimism and conveying good quality information to potential applicants is a key parameter to improving recruitment in trials [10]. Preceding studies have recorded the willingness of participants to be involved in COVID-19 vaccine trials; in studies in France and Jordan, 47.6% (1552/3259) and 36.1% (465/1287) of the participants, respectively, were willing to participate in COVID-19 vaccine trials in 2020 [11,12]. Another study conducted in Uganda that targeted health care workers and their supporting staff (non-health care professionals) across 3 hospitals recorded a 70.2% (n=461) willingness to participate in COVID-19 vaccine trials [13]. This raised an uncertainty whether health literacy or unfamiliarity with a health environment affects judgment, trust, and willingness to participate in COVID-19 vaccine trials in different community environments and geographical areas.

Reaching out to potential volunteers is one of many steps. Once presented with the opportunity to participate in trials, whether that be vaccine trials or clinical trials, one is troubled by the possibility of experiencing unpleasant symptoms and the consumption of time for participation, among other concerns, as well as mistrust in pharmaceutical companies [12]. Collecting data on patients’ thoughts, opinions, and beliefs across Europe may reveal effective methods for promoting volunteer registries and clinical research equity among diverse communities [14-16]. The objectives of this study are to assess European PAG members’ perspectives regarding vaccine trial participation along with their willingness to register in the Volunteer Registry [1,10]. Both are crucial factors prioritized by the VACCELERATE consortium [17]. Overall, the outcomes of this survey include the promotion of the VACCELERATE Volunteer Registry and ensuring the effective delivery of its work.

## Methods

### Study Design and Population

A standardized survey was designed to adhere to national bioethics’ requirements for each participating country and its target population including adult patients who are members of PAGs. The survey was transferred to an online format in collaboration with a consulting company (Conread Research Bureau Ltd) and was translated to English, Dutch, English, French, German, Greek, Italian, Spanish, and Swedish by the respective National Coordinator (NC) team of VACCELERATE. A list of PAGs per country was created, along with the appropriate contact details, by the task force undertaking this project with the guidance and support of the external consulting company and the respective VACCELERATE NC team.

### Data Collection

An official invitation letter was disseminated to PAGs via email between March 9, 2022, and May 16, 2022, together with the link to the online survey (see Multimedia Appendix 1). As the aim was to record PAG members’ willingness to register at the VACCELERATE Volunteer Registry [15,18], a description of the VACCELERATE program was provided at the beginning of the survey, followed by a question that asked whether participants were willing to join the registry.

Weekly reminders were sent via email to the NC or PAGs directly to increase responsiveness and engagement. All participants could exit the survey at any time they wished without completing it. Incomplete surveys (or without a signed consent form) were excluded from the final anonymous data analysis.

The collected data from the survey (Multimedia Appendix 1) included sociodemographic characteristics, prior COVID-19 vaccination status, history of SARS-CoV-2 infection, the source participants turn to for knowledge or information on health developments, and information regarding participants’ perceptions of important criteria for participating in vaccine trials. The questions posed were also an effort to understand communities’ and patients’ concerns regarding vaccine trials. This information could assist VACCELERATE investigators in designing and recruiting for future trials.

### Statistical Analysis

All results present the frequency at which a response was selected, in percentages. For the ordinal data, a Likert rating scale was used with the following options per characteristic:

1. Importance/relevance (1. Not at all important, 2. Somewhat not important, 3. Neither important nor unimportant, 4. Somewhat important, 5. To a great extent/extremely important)
2. Optimistic/pessimistic (1. Very negative, 2. Negative, 3. Neither negative nor positive—neutral, 4. Positive, 5. Very positive or 1. Very bad, 2. Bad, 3. Neither bad or good, 4. Good, 5. Very good, 6. Not available [N/A])

The percentages of responses for each of these selections were calculated. A score was established by calculating the dispersal of responses across the Likert scale (eg, the average between responses 1 and 5). Analysis was performed using SPSS v26 (IBM Corp).

For bivariate analysis and multivariate analysis, we used logistic regression analysis, as we wanted to calculate the effect of the independent variable on the binary dependent variable adjusted for confounders (ie, variables present that affect the variables under study and thus not allowing the results to mirror the real association between the dependent and independent variables). In particular, logistic regression analysis was performed to ascertain the effects of all demographic variables on the likelihood that a participant would be willing to participate in the VACCELERATE Volunteer Registry.

### Ethical Considerations

PAG members were requested to access a web link where they would find an electronic version of the survey and complete the questions honestly. To ensure all responders fully understood the objectives of the survey, the concept of anonymity, and data safety, the survey was provided in 9 different languages. The survey was approved by the Cyprus National Bioethics Committee (EEBK ΕΠ 2021.01.118) and other bioethics committees from the participating countries according to the national recommendations (Spain, Italy). Only responses with positive informed consent were processed, and no compensation was provided to responders. All records were anonymous and stored on a password-protected computer at Conread Research Bureau Ltd.

## Results

### General Characteristics

**Figure 1:**
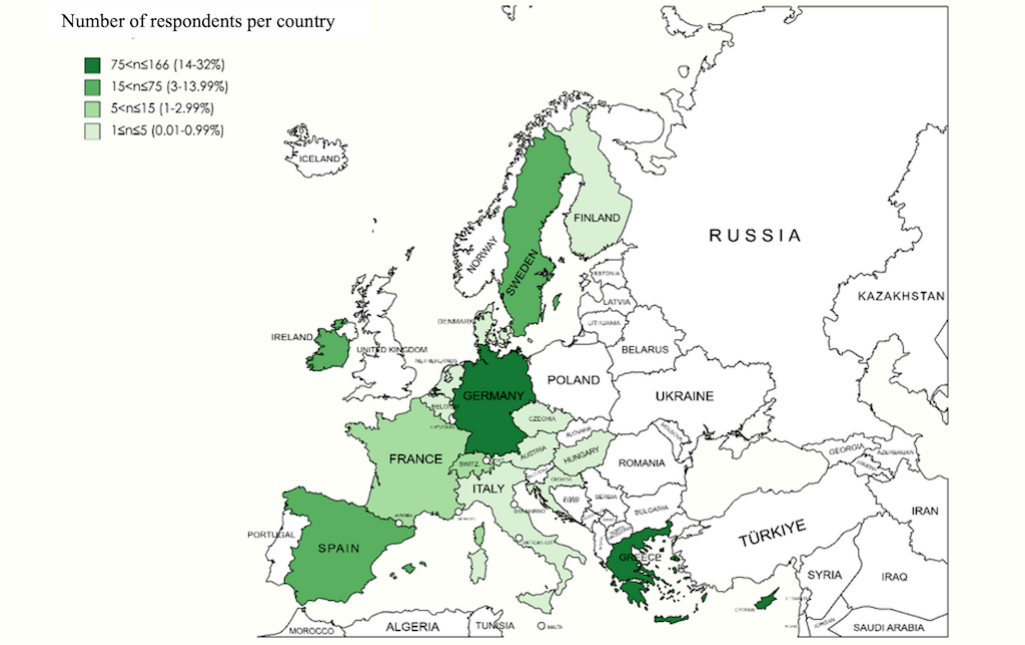
Illustration created using MapChart indicating the responses based on the residence country of the respondents (Belgium, Cyprus, Denmark, France, Germany, Greece, Ireland, Italy, Spain, and Sweden) for the VACCELERATE patient advocacy groups (PAGs) survey; although all the respondents’ nationalities belonged to the 10 European countries where the online anonymous questionnaire was circulated, registered responses from other countries resulted from differing countries of residence (created with MapChart [19], which is published under Creative Commons Attribution-ShareAlike 4.0 International License [20]).

A total of 520 responses were recorded through PAGs across the European region, as represented by a choropleth map (Figure 1) with the highest response rates recorded from Germany (165/520, 31.7%), Cyprus (149/520, 28.7%), and Greece (76/520, 14.6%). The fewest responses were collected from Denmark, Finland, Czechia, and Croatia (all had 1 response). Responses were obtained from PAG members from Belgium, Cyprus, Denmark, France, Germany, Greece, Ireland, Italy, Spain, and Sweden; some responses were obtained from other countries depending on the location of the respondents’ current residence.

Sociodemographic data showed higher participation rates by women (323/520, 62.1%). Regarding the age distribution (<29 years, 30-39 years, 40-49 years, 50-59 years, 60-69 years, and ≥70 years), most responses were collected from those aged 50-59 years (151/520, 29%). Regarding educational status, the greatest proportion of respondents had a postgraduate or doctorate degree (200/520, 38.5%), of whom 56% (112/200) reported that they predominantly consult news media for advice on health developments, while 55.5% (111/200) seek out physicians’ advice or even official international health organization websites and media (111/200, 55.5%). Individuals with a primary school education (106/168, 63.1%) mostly consult their doctors, while they tend to avoid social media (22/168, 13.1%). Last, undergraduates (89/146, 61%) rely on news media for advice on health developments.

Until May 2022, 71% (369/520) of PAG members were vaccinated with 3 doses of a COVID-19 vaccine, while 13.7% (71/520) had completed a fourth dose. Regarding the participants’ self-reported diagnosis of a COVID-19 infection, 37% (10/27; the highest percentage among participants with a self-reported disease group) of individuals with a confirmed COVID-19 diagnosis had “chronic cardio and pulmonary diseases”; however, an even higher percentage (10/21, 48%) of participants with a confirmed COVID-19 infection did not wish to state if they had an illness.

Table 1 depicts the self-reported chronic diseases based on stratification by the 2 main age groups, namely Age Group 1 and Age Group 2. The former includes 6 age subcategories (<29 years, 30-39 years, 40-49 years, 50-59 years, 60-69 years, ≥70 years), while the latter comprises 2 age subcategories (18-59 years, ≥60 years). For Age Group 1, those aged ≥70 years had the highest percentage of “All cancer conditions” (8/38, 21%), while those aged 50 years to 59 years had the lowest percentage (6/152, 4%). The highest and lowest percentages of “Rare diseases” were present for those aged 30 years to 39 years (24/88, 27%) or 60 years to 69 years (13/106, 12.3%). Finally, “Other diseases” were present the most for those aged 50 years to 59 years (94/152, 61.8%) and the least for those aged ≥70 years (15/38, 40%). On the other hand, in Age Group 2, the highest disease category for both age subgroups (18-59 years and ≥60 years) was “Other diseases,” at 53.2% (200/376) and 50.7% (73/144), respectively.

**Table 1:** Participants’ self-reported chronic illnesses and diseases or conditions stratified by Age Groups 1 and 2.

Chronic illness or disease	Age Group 1 (n_F_=520)							Age Group 2 (n_F_=520)	
	<29 years (n=40), n (%)	30-39 years (n=88), n (%)	40-49 years (n=96), n (%)	50-59 years (n=152), n (%)	60-69 years (n=106), n (%)	≥70 years (n=38), n (%)	18-59 years (n=376), n (%)		≥60 years (n=144), n (%)
All cancer conditions^a^	2 (5)	10 (11.4)	6 (6.3)	6 (4)	9 (8.5)	8 (21.1)	22 (5.9)		17 (11.8)
Rare diseases^b^	7 (17.5)	24 (27.3)	19 (19.8)	30 (19.7)	13 (12.3)	8 (21.1)	79 (21)		22 (15.3)
Immunosuppression^c^	3 (7.5)	9 (10.2)	14 (14.6)	15 (9.9)	15 (14.2)	2 (5.3)	41 (10.9)		17 (11.8)
Chronic cardio and pulmonary diseases	2 (5)	3 (3.4)	8 (8.3)	2 (1.3)	8 (7.6)	4 (10.5)	15 (4)		12 (8.3)
Other diseases^d^	22 (55)	40 (45.5)	43 (44.8)	94 (61.8)	58 (54.7)	15 (39.5)	200 (53.2)		73 (50.7)
I do not wish to state the chronic disease I have	4 (10)	2 (2.3)	6 (6.3)	5 (3.3)	3 (2.8)	1 (2.6)	19 (5.1)		3 (2)

^a^Hematological and solid cancers.

^b^A broad number of conditions.

^c^Acquired HIV or genetic-immunodeficiencies.

^d^Other diseases as stated by the respondents themselves included the following: rheumatoid arthritis, Parkinson disease, osteoarthritis, diabetes, celiac disease, ulcerative colitis, rheumatism, stroke, arrhythmias, hepatitis, systemic lupus erythematosus, psoriasis, ankylosing spondyloarthropathy, peripheral spondyloarthropathy, multiple sclerosis, and asthma, among others.

### Clinical Trial Awareness and Participation

Of the 520 respondents, 93.1% (484/520) claimed to be aware of the term “clinical trial,” of whom 22.5% (109/484) reported to have previously participated in clinical trials (Table 2). Regarding their rating of their experiences, having the ability to give feedback about the clinical trial process received the poorest rating (mean 3.29 out of 5). The highest rating was given for the level of information received while participating in the trial. None of the responses received a full rating. All 520 respondents were also asked to rate a list of criteria based on how important each criterion was in affecting their participation in clinical trials (Table 3). The criterion rated as “Extremely important” by the highest number of participants (386/520, 74.2%) was understanding the risks involved in one’s participation in such trials, followed by “Giving my consent after being fully informed on the process and have all my questions answered” (369/520, 71%). Having family or friends who had previously taken part in a vaccine clinical trial was an inadequate component for persuading one to participate or not participate in clinical trials (51/520, 9.8% voted it as “Extremely important”).

**Table 2:** From the 520 patient advocacy group (PAG) members who responded to the survey, 109 respondents had participated in clinical trials in the past and rated their experiences.

Respondents who had participated in clinical trials before (n=109)	Rating (1 to 5), n (%)						Mean rating (1 to 5)
	Very bad (1)	Bad (2)	Neutral (3)	Good (4)	Very good (5)		
Evaluation of the first set of information received (n=98)	1 (1)	7 (7.1)	17 (17.4)	38 (38.8)	35 (35.7)	4.01	
Information about the level of risk you took while taking part (n=100)	1 (1)	4 (4)	19 (19)	32 (32)	44 (44)	4.14	
The level of information you received (n=101)	0	10 (9.9)	19 (18.8)	42 (41.6)	30 (29.7)	3.91	
Ability to give your feedback about the clinical trial process (n=96)	6 (6.3)	20 (20.8)	24 (25)	32 (33.3)	14 (14.6)	3.29	
Regular communication with the medical team (n=99)	7 (7.1)	11 (11.1)	20 (20.2)	26 (26.3)	35 (35.4)	3.72	
Distance to/from the clinical site (n=99)	5 (5.1)	18 (18.2)	21 (21.2)	41 (41.4)	14 (14.1)	3.41	

**Table 3:** Importance of factors influencing participation in clinical trials, according to responders’ perceptions (n=520).

Criteria for clinical trial participation	Rating (1 to 5), n (%)						Mean rating (1 to 5)^a^
	Not at all important (1)	Somewhat not important (2)	Neither (3)	Somewhat important (4)	Extremely important (5)		
Understanding the purpose of the trial and how it will benefit others now and in the future	31 (6)	6 (1.2)	48 (9.2)	85 (16.4)	350 (67.3)	4.38	
Understanding the risks involved	31 (6)	7 (1.4)	33 (6.4)	63 (12.1)	386 (74.2)	4.47	
Understanding what the benefits will be from my participation	34 (6.5)	9 (1.7)	47 (9)	132 (25.4)	298 (57.3)	4.25	
Giving my consent after being fully informed on the process and have all my questions answered	34 (6.5)	6 (1.2)	30 (5.8)	81 (15.6)	369 (71)	4.43	
Being convinced that my personal data is fully protected	26 (5)	21 (4)	62 (11.9)	118 (22.7)	293 (56.4)	4.21	
Being convinced that my participation will be free of any financial cost	22 (4.2)	13 (2.5)	55 (10.6)	134 (25.8)	296 (56.9)	4.29	
The attitude of the researchers/physicians	24 (4.6)	5 (1)	39 (7.5)	108 (20.8)	344 (66.2)	4.43	
Getting to know/meet others who will take part in the same trial	66 (12.7)	63 (12.1)	174 (33.5)	122 (23.5)	95 (18.3)	3.23	
Having family or friends who have previously taken part in a clinical trial	150 (28.9)	86 (16.5)	171 (32.9)	62 (11.9)	51 (9.8)	2.57	

^a^Calculated by multiplying the absolute number of respondents with each rating, then the “grand total” was divided by the number of total respondents (n=520).

Participants were asked to evaluate the importance of each of the following 9 statements in their decision to take part in clinical trials: (1) understanding the purpose of the trial and how it will benefit others now and in the future, (2) understanding the risks involved, (3) understanding what the benefits will be from my participation, (4) giving my consent after being fully informed on the process and have all my questions answered, (5) being convinced that my personal data is fully protected, (6) being convinced that my participation will be free of any financial cost, (7) the attitude of the researchers/physicians, (8) getting to know/meet others that will take part in the same trial, (9) having family or friends who have previously taken part in a vaccine trial.

According to the regression model analysis for each of the aforementioned statements, the respondents from Northern European countries were (1) 4.484 (95% CI 2.618-7.962) times more likely to consider statement 1 (understanding the purpose of the trial and how it will benefit others now and in the future) as important for their decision, (2) 4.425 (95% CI 2.481-7.874) times more likely to consider statement 2 (understanding the risks involved) as important for their decision, (3) 1.838 (95% CI 1.164-2.907) times more likely to consider statement 3 (understanding what the benefits will be from my participation) as important for their decision (women were 1.628 [95% CI 1.041-2.547] times more likely to consider this statement as important for their decision), (4) 3.46 (95% CI 2.00-5.99) times more likely to consider statement 4 (giving my consent after being fully informed on the process and have all my questions answered) as important for their decision, (5) 2.551 (95% CI 1.639-3.968) times more likely to consider statement 5 (to be convinced that my personal data is fully protected) as important for their decision (women were 2.116 [95% CI 1.384-3.236] times more likely to consider it as important for their decision). (6) 1.773 (95% CI 1.127-2.786) times more likely to consider statement 6 (being convinced that my participation will be free of any financial cost) as important for their decision, (7) 2.470 (95% CI 1.444-4.226) times more likely to consider statement 7 (the attitude of the researchers/physicians) as important for their decision (people younger than 70 years were 3.481 [95% CI 1.586-7.642] times more likely to consider this statement as important for their decision). Respondents younger than 70 years were (8) 3.33 (95% CI 1.35-8.20) times more likely to consider statement 8 (getting to know/meet others that will take part in the same trial) as important for their decision. Respondents from Southern European countries were (9) 2.96 (95% CI 1.90-4.63) times more likely to consider statement 9 (having family or friends who have previously taken part in a vaccine trial) as important for their decision.

### VACCELERATE Volunteer Registry

The score for a willingness to participate in the Volunteer Registry was 4 “Positive” for 25.6% (133/520) of the participants and 5 “Very positive” for 9% (47/520) of the participants (Table 4). The highest score for a willingness to participate was 3.24 for those aged 60 years to 69 years. The perspectives of participants regarding registration (per country) in the Volunteer Registry is presented in Table 5, while motives for participating in the Volunteer Registry did not vary among the sexes (Table 6).

**Table 4:** Insight into the willingness of the 520 respondents to participate in the VACCELERATE Volunteer Registry sorted by age group.

Age Group 1	Willingness to participate (1-5), n (%)						Mean rating (1 to 5)
	Very negative (1)	Negative (2)	Neither negative nor positive (3)	Positive (4)	Very positive (5)		
Total sample	32 (6.2)	72 (13.9)	236 (45.3)	133 (25.6)	47 (9)	3.18	
≤29 years (n=40)	3 (7.5)	5 (12.5)	15 (37.5)	14 (35)	3 (7.5)	3.23	
30-39 years (n=88)	8 (9.1)	12 (13.6)	36 (40.9)	27 (30.7)	5 (5.7)	3.10	
40-49 years (n=96)	8 (8.3)	15 (15.6)	44 (45.8)	17 (17.7)	12 (12.5)	3.10	
50-59 years (n=152)	7 (4.6)	19 (12.5)	76 (50)	36 (23.7)	14 (9.2)	3.20	
60-69 years (n=106)	5 (4.7)	14 (13.2)	48 (45.3)	29 (27.4)	10 (9.4)	3.24	
≥70 years (n=38)	1 (2.6)	7 (18.4)	17 (44.7)	10 (26.3)	3 (7.9)	3.18	

**Table 5:** Patient advocacy group (PAG) members’ (n=520) willingness to participate in the VACCELERATE Volunteer Registry, by country of residence.

Country of residence	Willingness to participate (1-5), n (%)						Mean rating (1-5)
	Very negative (1)	Negative (2)	Neither negative nor positive (3)	Positive (4)	Very positive (5)		
Germany (n=165)	7 (4.2)	23 (13.9)	73 (44.2)	47 (28.5)	15 (9.1)	3.24	
Cyprus (n=149)	15 (10.1)	24 (16.1)	67 (45)	35 (23.5)	8 (5.4)	2.98	
Greece (n=76)	5 (6.6)	15 (19.7)	39 (51.3)	9 (11.9)	8 (10.5)	3.00	
Ireland (n=36)	0	2 (5.6)	16 (44.4)	10 (27.8)	8 (22.2)	3.67	
Spain (n=33)	3 (9.1)	3 (9.1)	15 (45.5)	10 (30.3)	2 (6.1)	3.15	
Sweden (n=19)	0	1 (5.3)	10 (52.6)	6 (31.6)	2 (10.5)	3.47	
France (n=13)	1 (7.7)	2 (15.4)	3 (23.1)	5 (38.5)	2 (15.4)	3.38	
Other^a^ (n=29)	1 (3.5)	2 (6.9)	13 (44.8)	11 (37.9)	2 (6.9)	3.38	

^a^Belgium, Croatia, Czechia, Denmark, Finland, Hungary, Italy, Luxemburg, The Netherlands, and Switzerland; all with the lowest response rates.

**Table 6:** Of the respondents willing to participate in the VACCELERATE Volunteer Registry (n=180), reasons given for opting in, by gender.

Reasons	All respondents, n (%)	Men (n=77), n (%)	Women (n=103), n (%)
Help advance medical research	155 (86.1)	68 (88.3)	87 (84.5)
Receive payment upon my participation	26 (14.4)	12 (15.6)	14 (13.6)
I lost one of my own and want to help medical science advance to help people overcome their problems.	29 (16.1)	12 (15.6)	17 (16.5)
As a patient, I believe science would be greatly supported to enrich their data by my participation.	131 (72.8)	57 (74)	74 (71.8)
I (or someone close to me) had a pleasant experience while taking part in a clinical trial in the past.	14 (7.8)	8 (10.4)	6 (5.8)
I will be one of the first to get to know the medical advancements.	53 (29.4)	18 (23.4)	35 (34)
Other reasons	3 (1.7)	1 (1.3)	2 (1.9)

Countries in which participants were more willing to join the Volunteer Registry were Ireland (3.67/5.00), Sweden (3.47/5.00), and France (3.38/5.00; Table 5). The respondents who were both “positive” and “very positive” were then directed to a supplementary question to select a reason for choosing to join the Volunteer Registry. The most widely held reason was to “Help advance medical research” (155/180, 86.1%), and the greatest proportion of this response came from individuals aged <29 years or ≥70 years. The second most popular response, at 72.8% (131/280), was “As a patient, I believe science would be greatly supported to enrich their data by my participation.” An additional point of interest was the option to “Receive payment upon my participation,” which was recorded by 18.8% (24/128) of those aged 18 years to 59 years, whereas for those ≥60 years of age, the percentage was significantly lower, at only 3.9% (2/52).

The 104 survey participants who were unwilling to register in the Volunteer Registry were asked for justification. The 2 most frequently reported reasons across all countries for refusing were “Do not trust pharmaceutical companies/medical researchers/private or public companies” (27/104, 26%), followed by “Do not trust government agencies/services on health issues” (26/104, 25%). These responses were especially popular in the Mediterranean region (Cyprus and Greece), where the score was the lowest in terms of willingness to participate in the Volunteer Registry. Additional reasons why respondents were unwilling to participate in the Volunteer Registry included being “concerned about the safety of the vaccines (side effects),” “went through COVID-19 in the past and in no need of a vaccine, so no need to participate in clinical trials,” “I (or someone close to me) had an unpleasant experience while taking part in a clinical trial in the past,” being “concerned about the misuse of my personal data and invasion of my privacy,” “participating is against my religious beliefs,” and finally, “I consider human experimentation unethical.” The logistic regression model analysis showed that the European Region (north vs south) was a predictor variable for the patients’ willingness to register in the VACCELERATE Volunteer Registry (χ^2^_1_=9.009, *P*=.003). Respondents from Northern European countries were 1.725 (95% CI 1.206-2.468) times more likely to be willing to participate in the VACCELERATE Volunteer Registry.

## Discussion

### Principal Findings

The COVID-19 pandemic has shown the significance and importance of having people voluntarily participating in the development of new vaccines. The objectives of this effort were to approach PAG members across Europe through an online survey to assess their perspectives related to participation in vaccine trials despite their diagnoses. This study was also an opportunity to promote the Volunteer Registry to more diverse communities and measure patients’ willingness to register in the registry. VACCELERATE intends to approach individuals with morbidities or comorbidities with an objective to ensure inclusiveness and research equity as well as increase literacy about vaccine and clinical trials. Ultimately, VACCELERATE hopes to make participation in well-designed clinical trials a normal part of the scientific progress that leads to better medical care for all [21].

At the time of questionnaire preparation, only 3 COVID-19 vaccination doses were advocated across Europe; however, upon survey launch, a fourth dose had been introduced, which may explain the low percentage of the later dose recorded in the survey. The survey results also demonstrate that members of all educational backgrounds seek health advice from a doctor, emphasizing the importance of communicating with and training doctors on innovative medicine and clinical trials. Recruiting and accurately training the medical team to execute all aspects and stages of a clinical trial are also of high importance to instill confidence in and attract the attention of potential volunteers. Communication regarding any concerns a potential volunteer may have, a clear breakdown of all information regarding the medication or vaccine, description of any symptoms and procedures to be carried out, and volunteer support are all key factors a trial team should be able to deliver [9].

Trial investigators should prioritize the incorporation of benefits; communicate trial benefits (access to new drugs and vaccines) to potential volunteers; and ensure patients feel safe, supported, and, above all, well informed throughout the whole duration of a trial. This can be achieved by establishing trust and addressing knowledge deficits (eg, provide informative material in the patient’s native language and using simple terms) to prevent misinformation and uphold transparency. Communicating this information and establishing a network of active participants in clinical trials may be facilitated via collaboration with PAGs [22]. Furthermore, educating potential volunteers on the value of their participation and input in vaccine trials and how the pandemic’s impact on the community can be minimized may inspire further volunteering [23,24].

The most popular reason for opting to participate in the Volunteer Registry is to help advance medical research, an opinion that has been recorded in the past as a motivating factor for participation [23]. Potential volunteers may also be driven by the offer of potential personal benefit, a view which was not strongly supported by the PAG members of this study [24]. On the other hand, unwillingness to join the registry was attributable to participants’ mistrust in pharmaceutical companies, medical researchers, private or public companies, and governmental agencies [12]. Volunteering may also be affected by personal ideologies, past experiences, ethnicity, and religion, among other factors. Based on this study’s results, a significant difference in the willingness to participate in the registry has been observed between Northern and Southern European respondents. This observation needs to be further evaluated in the future.

Feedback on past participation in clinical trials involving a certain medicine showed that PAG members had an overall positive experience. Participants reported that, in terms of the quality and quantity, the information they received about the level of risk they were taking while participating in the trial was good overall. Still, 2 important aspects were lacking: offering volunteers the opportunity to provide feedback regarding their experience in the trial and providing opportunities for regular communication with the medical team. Both of these aspects may prevent participants from joining vaccine trial registries or participating in clinical trials, as these outcomes may affect the security and trust one feels in partaking in clinical trials. Therefore, the participants’ reactions indicate there is still room for improvement when designing and developing clinical trials in which support, inclusiveness, and safety of volunteers are prioritized.

### Limitations

Limitations were recognized during the execution of the study. The number of responses from women was significantly greater than that from men, raising concerns of gender disparities as well as limited generalizability. An additional factor that may have shaped these results is that a greater number of respondents had a higher level of education (38% of them had a postgraduate or doctorate degree). Finally, as most responses were collected from Germany, Greece, and Cyprus (Figure 1), there was deliberation as to whether the results can be considered representative of PAGs across Europe.

### Conclusions

This study exhibited and analyzed data from a Pan-European, online survey targeting adult individuals with chronic underlying conditions (patients) from the European region who are members of PAGs.

Despite the high percentage of participants acknowledging awareness of the term “clinical trials,” few individuals affirmed they were willing to participate in the Volunteer Registry due to a lack of trust in certain bodies involved in clinical trial conduct, concerns involving safety, or even religious beliefs. The results of this online survey represent only an initial indication of patient willingness to register in the Volunteer Registry and support that PAG members value advancements in medical research and clinical trials.

The information presented aids in interpreting issues and setbacks in existing registrations so that a plan may be constructed to improve future promotion, campaigns, and approach schemes for vaccine trials, in particular for PAG members. Overall, raising public awareness of the benefits of clinical trials and improving health literacy may increase participation in vaccine clinical trials. Careful planning and more thought need to be invested in designing trials to guarantee inclusiveness, equality, and strong support networks for groups such as PAGs.

## Appendix

Multimedia Appendix 1 Invitation letter.

## Abbreviations

PAG: patient advocacy group
NC: National Coordinator

## Data Availability

All data generated or analyzed during this study are included in this published article and its supplementary information files.
