# Supplementary material for: Perspectives of European Patient Advocacy Groups on Volunteer Registries and Vaccine Trials: VACCELERATE Survey Study
Source: JMIR Public Health Surveill. 2024 Apr 4;10:e47241. doi: 10.2196/47241 (PMC10996911; doi:10.2196/47241)
Supplement: Multimedia Appendix 1 [file publichealth_v10i1e47241_app1.docx]

## Appendices

**Appendix 1:** Invitation letter.

| Dear Sir/Madam  We are contacting **XXX**, as we are carrying out a pan-European EC funded project (**Horizon program 2020**), related to clinical trials & the development of new vaccines, the key objective of which is to improve the EU member states preparedness levels against future epidemics/pandemics. Additionally, a key challenge is also to provide the means to face COVID-19 vaccinations within public and chronic patients more effectively. The project is titled “VACCELERATE” and its findings will support countries and associations with expertise, & solutions to speed up existing and upcoming development programs for new vaccines and vaccination strategies. For more information about Vaccelerate, please click the button below. |
| --- |
| \| [CLICK FOR MORE INFO](https://conread.us20.list-manage.com/track/click?u=12e0c82f644c18ef6711f0661&id=ba847416f5&e=fb69723962) \| \| --- \| |
| Our survey is addressed to NGO’s, Leagues, members of Patient Advocacy Groups and also the general public.  The opinions of **XXX** **members** are very important to us, and we would appreciate it if you would be kind enough to support the effort by inviting your members to participate in this survey. Please feel free to forward this invite to your members and kindly ask them to click on the following link to fill in the survey. We would much appreciate your support: |
| \| [COMPLETE QUESTIONNAIRE](https://www.dooblocawi.com/client/Survey.aspx?Ticket=5XVAEMXA) \| \| --- \| |
| The survey will always remain anonymous and will take no more than 4-5 minutes to be completed by your members. Your contribution will be highly appreciated and communicated with special references during dissemination phase, while the project final insights can be shared among all project’s supporting organizations, should it become a requirement. Our survey will remain live for completion until end of April.  Should you have any queries, please do not hesitate contacting me directly at XXX or ++XXXXXXXX  Thank you in advance for your time and consideration and we are at your disposal in case you need any additional information. |

**Appendix 2:** Electronic form of the PAG questionnaire (English).

1. **Please select a language:**

English

Deutsch

Ελληνική

Italiano

Svenska

Français

Castellano

Nederlands

1. **VACCELERATE (https://vaccelerate.eu/) is the new Pan-European COVID-19 vaccine trial** network funded by the European Commission to support vaccine trials in Europe.

The VACCELERATE network has set up a Volunteer Registry for people interested to participate in vaccine trials. This registry is a new effort to promote inclusiveness and equality for every person in clinical trials. Therefore, the aim is to mobilize potential volunteers across the European region to express their interest for future participation in clinical trials. For more information, please visit: <https://vaccelerate.eu/volunteer-registry/>

The aim of this survey is

- to address the challenges of finding volunteers for clinical trials in patient groups in Europe.
- to deepen our knowledge about issues volunteers might have when deciding about participating in clinical trials
- to acknowledge the participants’ suggestions for trial participation improvement strategies

In addition, we would like to engage European patient advocacy groups in the process of informing and promoting the VACCELERATE Volunteer Registry and ask about their members’ willingness to register in the Volunteer Registry during this survey.

**I have read and fully understood the above information and I consent to the analysis of the anonymously collected data for the purpose of the present survey by VACCELERATE**.

Please tick the selected answer in the given box (only one answer allowed)

I consent

I do not consent

1. **Which chronic illness/disease do you have?**

Please tick the selected answer in the given box (only one answer allowed)

All cancer conditions (hematological and solid cancers)

Rare diseases (a broad number of conditions)

Immunosuppression (acquired HIV or genetic immunodeficiencies)

Chronic cardio and pulmonary diseases

Other diseases (Specify)

I do not wish to state the chronic disease I have

None

1. **Which medical association are you a member of?**

Please use lines to fill up a free-text answer

1. **In which of the following age groups do you belong?**

Please tick the selected answer in the given box (only one answer allowed)

15 years and younger

16-17 years

18-20 years

21-24 years

25-29 years

30-39 years

40-49 years

50-59 years

60-69 years

70-79 years

80 + years

1. **Country of residence**

Please tick the selected answer in the given box (only one answer allowed)

Austria

Belgium

Bulgaria

Croatia

Cyprus

Czechia

Denmark

Estonia

Finland

France

Germany

Greece

Hungary

Ireland

Italy

Latvia

Lithuania

Luxembourg

Malta

Netherlands

Poland

Portugal

Romania

Slovakia

Slovenia

Spain

Sweden

Other (Please specify)

1. **Residence Area**

Please tick the selected answer in the given box (only one answer allowed)

Urban

Rural

1. **Gender**

Please tick the selected answer in the given box (only one answer allowed)

Male

Female

Other (specify)

1. **Marital Status**

Please tick the selected answer in the given box (only one answer allowed)

Single

Married

Divorced

In a relationship

Widowed

1. **Are you a parent?**

Please tick the selected answer in the given box (only one answer allowed)

Yes

No

1. **Which of the following is your highest education level?**

Please tick the selected answer in the given box (only one answer allowed)

Did not attend school

Primary school

High School

Undergraduate degree

Post Graduate degree

Doctorate degree or equivalent level

Prefer not to say

1. **Which of the following best describes your employment status?**

Please tick the selected answer in the given box (only one answer allowed)

employed full-time (≥ 35h per week)

employed part-time (15 – 34h per week)

minimal employment (< 15h per week)

Student

unemployed

I prefer not to say

1. **Which of the following best describes your employment status?**

Please tick the selected answer in the given box (only one answer allowed)

Employed

Self-Employed

Unemployed

Student

Retired

Disabled/not able to work

I prefer not to say

1. **What is your nationality?**

Please tick the selected answer in the given box (only one answer allowed)

Austrian

Belgium

British

Croatian

Cypriot

Czech

Danish

Dutch

Estonian

Finnish

French

German

Greek

Hungarian

Italian

Irish

Latvian

Lithuanian

Luxembourgian

Maltese

Norwegian

Polish

Portuguese

Romanian

Russian

Slovakian

Slovenian

Spanish

Swiss

Swedish

Turkish

Other Nationality (Specify)

1. **What is your COVID-19 vaccination status?**

Please tick the selected answer in the given box (only one answer allowed)

1 dose

2 doses

3 doses

4 doses

Other (Specify)

I have not received any dose

1. **Have you already been diagnosed with COVID-19?**

Please tick the selected answer in the given box (only one answer allowed)

Yes

No

Not sure

I prefer not to say

1. **How did you face the disease when you went through it (COVID-19)?**

Please tick the selected answer in the given box (only one answer allowed)

Ι was hospitalized in a hospital / clinic (private or public)

I was taken care of at home with medicine prescribed by doctors

No medical treatment was necessary

I did not even realise I was going through COVID-19 / I was only found with high antibodies later one

I prefer not to say

1. **Which of the following are your primary sources of information regarding health developments / medical research / clinical trials / etc.?**

Please tick the selected answer in the given box (minimum: 1, maximum: 8)

Official international health organisation websites and media e.g., WHO, ECDC

Official government websites and media

News Media e.g., TV, radio, newspapers

Social Media e.g., Facebook, Twitter, Instagram

Medical journals

Word of mouth – friends & family

Doctor’s advice

Other (specify)

1. **Which one of these is your primary source of information regarding health/medical research/clinical trials/etc.?**

Please circle the selected answer (only one answer allowed)

Official international health organisation websites and media e.g., WHO, ECDC.

Official government websites and media

News Media e.g., TV, Radio, Newspapers

Social Media e.g., Facebook, Twitter, Instagram

Medical journals

Word of mouth – friends & family

Doctor’s advice

Other (specify)

1. **With this question, we would like to better understand your attitude towards life itself. Please answer Yes or No to the following statements/phrases below.**

For each topic, please circle the number which represents your answer.

1. Yes

2. No

I worry more than an average person

2

1

I am an optimistic person

2

1

I am religious

2

1

I am regularly updated via the news

2

1

I am ecologically friendly/environmentally aware

2

1

I consider myself socially sensitive

2

1

1. **Are you aware of the term Clinical Trials?**

Please tick the selected answer in the given box (only one answer allowed)

Yes, I am aware of the term "Clinical Trials"

No, I am not aware of it

1. **Have you ever participated in any Clinical Trial?**

Please tick the selected answer in the given box (only one answer allowed)

Yes, I have participated in Clinical Trials before

No, I have not participated in Clinical Trials before

1. **Was your participation in the Clinical Trial related to medicine or vaccine (of any kind)?**

Please tick the selected answer in the given box (only one answer allowed)

It was related to a medicine

It was related to a vaccine (of any kind)

Both – medicine & vaccine (of any kind)

Other (specify)

1. **How many times have you participated in any Clinical Trial in the past?**

Please tick the selected answer in the given box (only one answer allowed)

One time only

Twice

Three times

Four times

More than four times

1. **In which Phase(s) of Clinical Trial(s) did you participate?**

Please tick the selected answers in the given box (minimum: 1, maximum: 5)

Phase I

Phase II

Phase III

Phase IV

I do not know / I prefer not to say

1. **How would you evaluate your overall experience of participating in a/the Clinical Trial(s)?**

For each topic, please select the number which represents your answer.

1. Very bad

2. Bad

3. Neither good or bad

4. Good

5. Very good

6. N/A

Evaluation of the overall experience in participating in Clinical Trials

| 1 | 2 | 3 | 4 | 5 | 6 |
| --- | --- | --- | --- | --- | --- |

1. **How would you evaluate each of the following criteria based on your overall experience with participating in phases I/II/III/IIII of the Clinical Trials?**

For each topic, please circle the number which represents your answer.

1. Very Bad

2. Bad

3. Neither Bad or Good

4. Good

5. Very Good

6. N/A

Evaluation of the first set of information received

| 1 | 2 | 3 | 4 | 5 | 6 |
| --- | --- | --- | --- | --- | --- |

Information about the level of risk you took while taking part

| 1 | 2 | 3 | 4 | 5 | 6 |
| --- | --- | --- | --- | --- | --- |

The level of information you received

| 1 | 2 | 3 | 4 | 5 | 6 |
| --- | --- | --- | --- | --- | --- |

The ability to give your feedback with regard to the clinical trial process (before, during and at the end of the process)

| 1 | 2 | 3 | 4 | 5 | 6 |
| --- | --- | --- | --- | --- | --- |

Regular communication with the medical team over the course of the study

| 1 | 2 | 3 | 4 | 5 | 6 |
| --- | --- | --- | --- | --- | --- |

Distance to/from the clinical site

| 1 | 2 | 3 | 4 | 5 | 6 |
| --- | --- | --- | --- | --- | --- |

**Clinical trials are research studies performed in people that are aimed at evaluating a medical, surgical, or behavioral intervention. They are the primary way that researchers find out if a new treatment, like a new drug or diet or medical device (for example, a pacemaker) is safe and effective in people.**

1. **How important is each of the following in your decision to take part in clinical trials?**

For each topic, please circle the number which represents your answer.

1. Not at all important

2. Somewhat not important

3. Neither important nor unimportant

4. Somewhat important

5. To a great extend / extremely important

Understanding the purpose of the trial and how it will benefit others now and in the future

| 1 | 2 | 3 | 4 | 5 |
| --- | --- | --- | --- | --- |

Understanding the risks involved

| 1 | 2 | 3 | 4 | 5 |
| --- | --- | --- | --- | --- |

Understanding what the benefits will be from my participation

| 1 | 2 | 3 | 4 | 5 |
| --- | --- | --- | --- | --- |

Giving my consent after being fully informed on the process and have all my questions answered

| 1 | 2 | 3 | 4 | 5 |
| --- | --- | --- | --- | --- |

To be convinced that my personal data is fully protected

| 1 | 2 | 3 | 4 | 5 |
| --- | --- | --- | --- | --- |

Το be convinced that my participation will be free of any financial cost

| 1 | 2 | 3 | 4 | 5 |
| --- | --- | --- | --- | --- |

The attitude of the researchers/physicians

| 1 | 2 | 3 | 4 | 5 |
| --- | --- | --- | --- | --- |

Getting to know /meet others that will take part in the same trial

| 1 | 2 | 3 | 4 | 5 |
| --- | --- | --- | --- | --- |

Having family or friends who have previously taken part in a Clinical Trial

| 1 | 2 | 3 | 4 | 5 |
| --- | --- | --- | --- | --- |

1. **Would you be willing to participate in the VACCELERATE Volunteer Registry for vaccine trials?**

For each topic, please circle the number which represents your answer.

1. very negative

2. negative

3. neither negative nor positive - neutral

4. positive

5. very positive

Willingness level for participating in a Volunteer Registry for clinical trials

| 1 | 2 | 3 | 4 | 5 |
| --- | --- | --- | --- | --- |

1. **What are your reasons for opting to participate in the VACCELERATE Volunteer Registry for clinical trials of approved or pre-approved vaccines by the EMA (European Medicines Agency)?**

**Express yourself with as many details as possible:**

Please tick the selected answers in the given box (minimum: 1, maximum: 7)

I want to help advance Medical Research

I will receive payment upon my participation

I lost a family member/close friend and I would like to help medical science get as advanced as possible to help people/patients overcome their problems

As a patient, I believe that science would be greatly supported to enrich their data by my participation

I (or someone close to me) had a pleasant experience while taking part in a clinical trials in the past

I will be one of the first to get to know the medical advancements

Other reasons (specify)

1. **What are your reasons for taking a negative position on your participation in the VACCELERATE Volunteer Registry?**

Please tick the selected answers in the given box (minimum: 1, maximum: 9)

I (or one of my close persons) had an unpleasant experience taking part in a clinical trial in the past

Ι am not getting vaccinated as I am concerned about the safety of the vaccines (side-effects)

I am concerned about the misuse of my personal data and invasion of my privacy

I had COVID-19 in the past and I don’t need a vaccine, so there is no need to take part in such clinical trials

I don’t trust pharmaceutical companies / medical researchers / private or public companies

In general, I don't trust government agencies / services on health issues etc.

Participating is against my religious beliefs

I consider human experimentation unethical

Other reasons (specify)

**Express yourself with as many details as possible:**

**Thank you for your cooperation!**
